# Supplementary material for: In Silico Identification of Anti-SARS-CoV-2 Medicinal Plants Using Cheminformatics and Machine Learning
Source: Molecules. 2022 Dec 26;28(1):208. doi: 10.3390/molecules28010208 (PMC9821958; doi:10.3390/molecules28010208)
Supplement: Supplementary file 1 [file molecules-28-00208-s001.zip › molecules-2030956-supplementary/Supplementary Materials.pdf]

Supplementary Figure S1

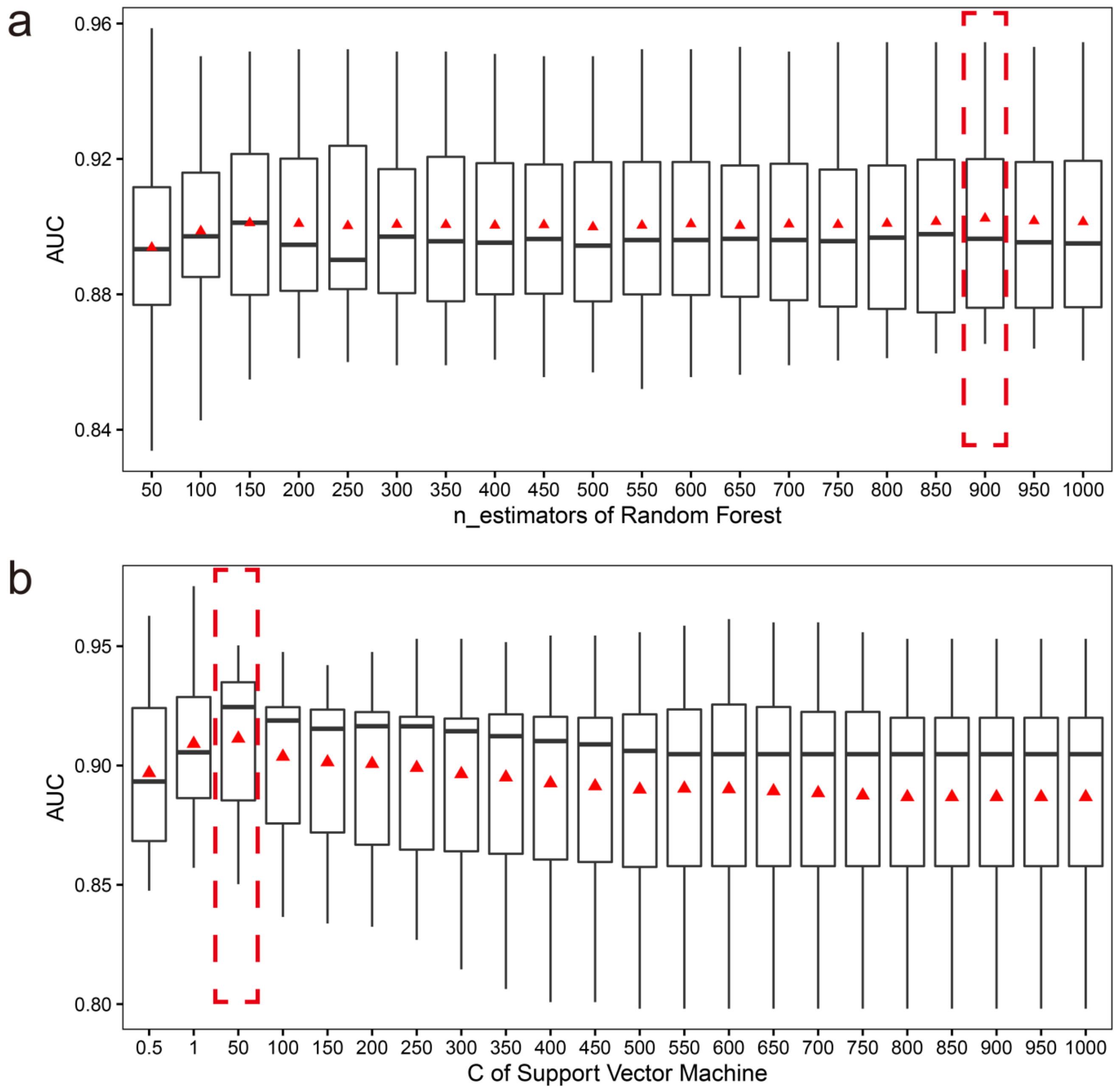

Supplementary Figure S1. Parameter optimization of the RF and SVM models. (a). The optimal parameter n_estimators of the RF model was determined according to the highest mean AUC of 10-fold cross validation. (b). The optimal parameter C of the SVM model was determined according to the highest mean AUC of 10-fold cross validation.

Supplementary Figure S2

a

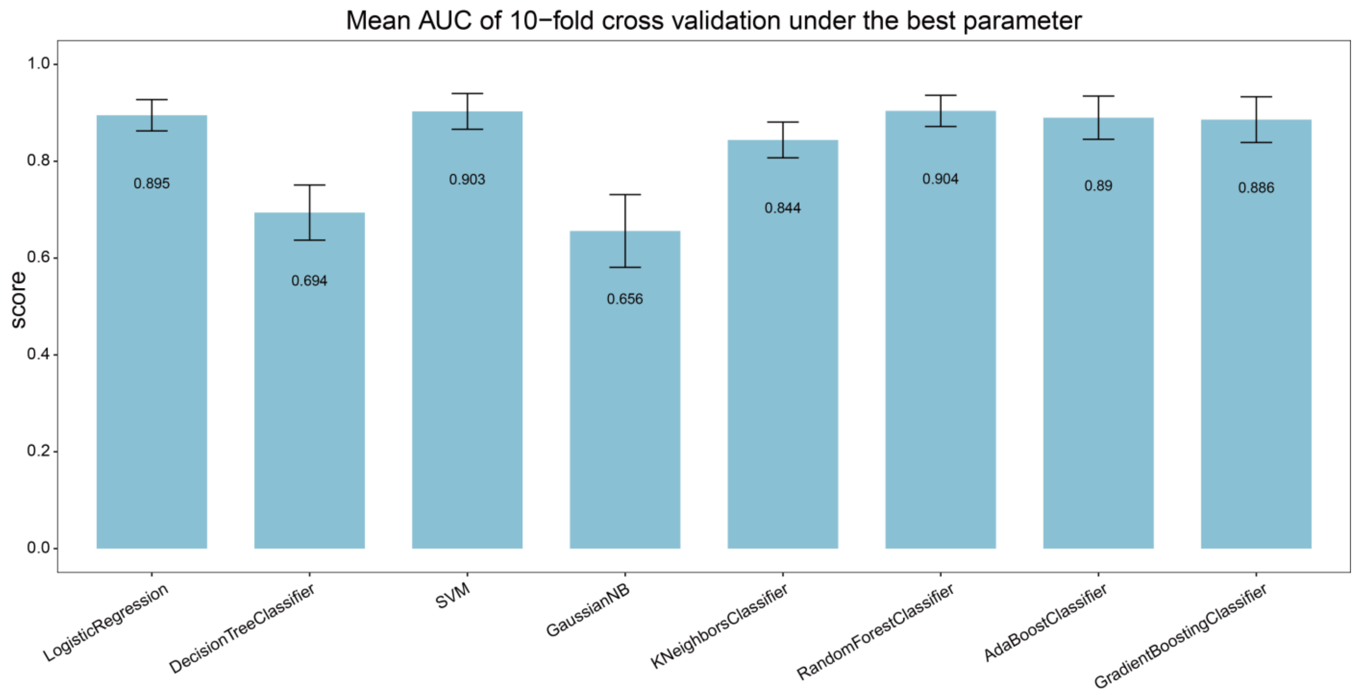

b

Method	Parameter	Mean AUC
LogisticRegression	C=0.5	0.895
DecisionTreeClassifier	Default parameters	0.694
Support Vector Machine	C=50	0.903
GaussianNB	Default parameters	0.656
KNeighborsClassifier	n_neighbors=10	0.844
RandomForestClassifier	n_estimators=900	0.904
AdaBoostClassifier	n_estimators= 1000	0.890
GradientBoostingClassifier	n_estimators= 400	0.886

Supplementary Figure S2. The AUC value of different machine learning classification methods for benchmark datasets. (a). Bar plots show the mean AUC values of 10-fold cross validation derived from different machine learning classification methods for benchmark datasets. The error bars represent the standard deviations. (b). Optimal parameters and mean AUC value of different machine learning classification methods for benchmark datasets.

Supplementary Figure S3

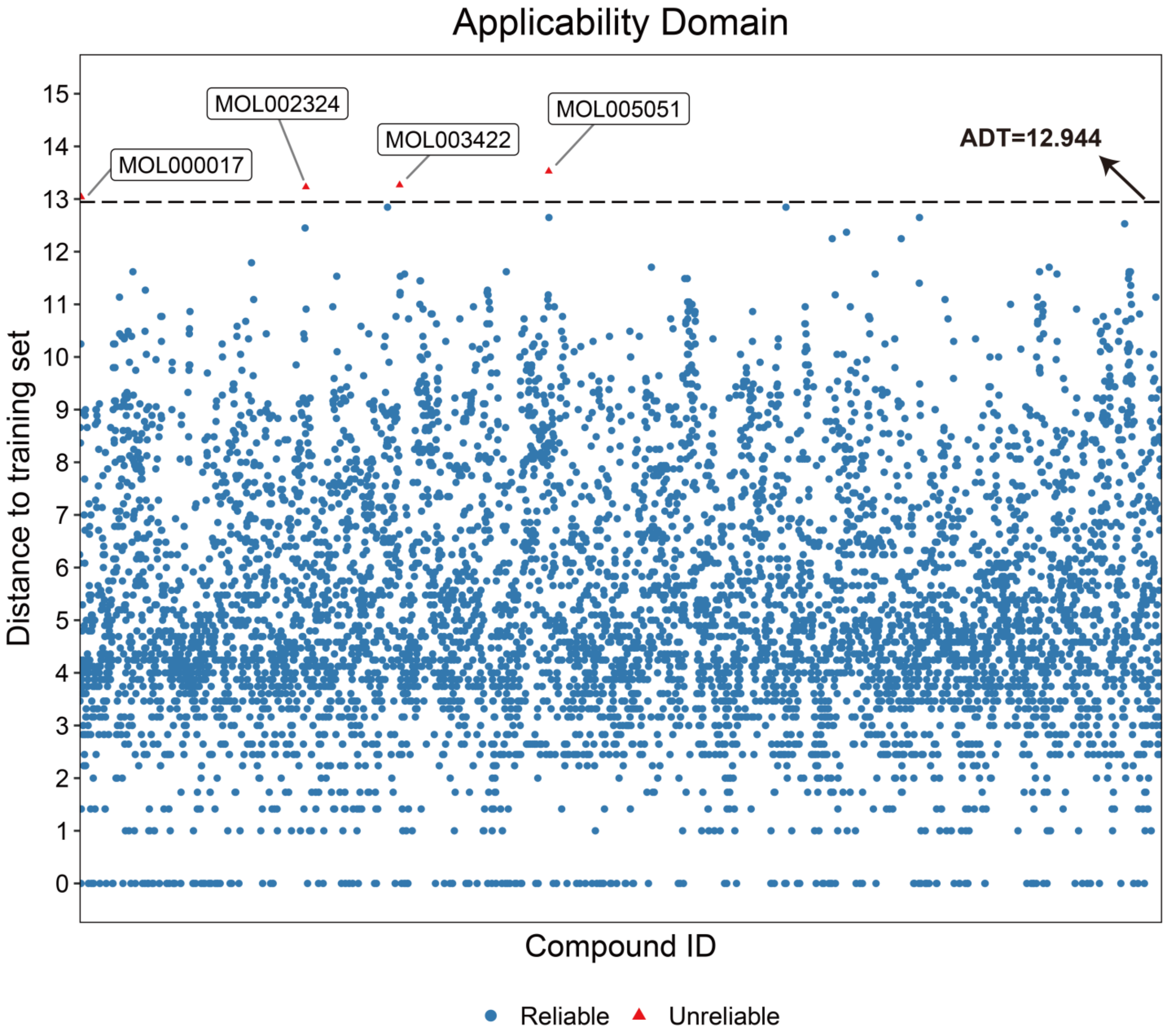

Supplementary Figure S3. The scatter plot indicates whether the TCMSP compounds is inside the applicability domain (AD) of our models. Each dot or triangle represents a compound. blue dot indicate that the compound is inside the AD. Red triangle indicate that the compound is outside the AD. The applicability domain threshold (ADT) is 12.944.

Supplementary Figure S4

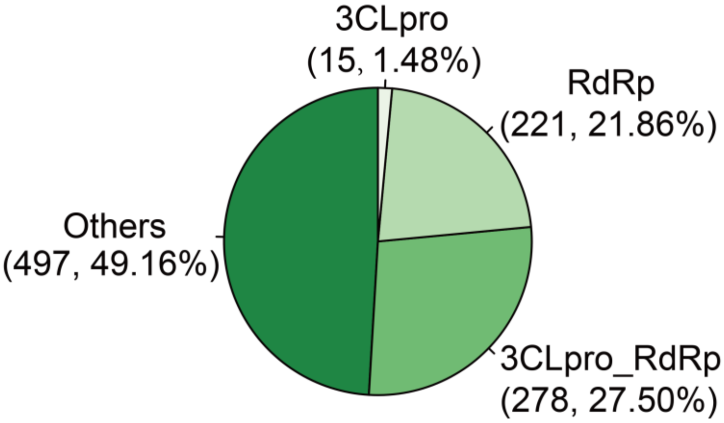

Supplementary Figure S4. The pie chart showing the number of compounds against RdRp, 3CLpro and other proteins based on the docking results. The RNA-dependent RNA polymerase (RdRp) and 3C-like protease (3CLpro) encoded by SARS-CoV-2 genome play important roles in the viral life cycle and are considered the most promising targets for drug discovery against SARS-CoV-2. We used AutoDock Vina (v1.1.2) to conduct virtual screening of 1011 active anti-SARS-CoV-2 compounds against RdRp and 3CLpro proteins, respectively. The 3D structures of the RdRp (PDB ID: 7BV2) and 3CLpro (PDB ID: 7VH8) were downloaded from the Protein Data Bank. The ligand center of the complex is used as the docking center. 3CLpr inhibitor PF-07321332 (-7.0 kcal/mol) and RdRp inhibitor Remdesivir (-5.3 kcal/mol) were selected as positive controls for RdRp and 3CLpro proteins, respectively. The docking results showed that 15 compounds specifically docked to the 3CLpro protein, 221 compounds specifically docked to the RdRp protein, and 278 compounds could dock to both the 3CLpro and RdRp proteins.
